# The burden and management strategies of hypertensive crisis in adult patients presenting to emergency departments of district and regional hospitals in Sub-Saharan Africa

**DOI:** 10.1186/s40885-023-00251-8

**Published:** 2023-10-01

**Authors:** Said S. Kilindimo, Ahmed Abdulkarim, Alphonce N. Simbila, Raynald Harrison, Lucy Shirima, Farida Abdallah, Aliasghar G. Mukhtar, Juma Mfinanga, Joseph Saika, Emanuel Kisanga, Hendry R. Sawe

**Affiliations:** 1 Emergency Medicine Department, Muhimbili University of Health and Allied Sciences, P.O Box 65001, Dar es Salaam, Tanzania; 2 Emergency Medicine Department, Muhimbili National Hospital, Dar es Salaam, Tanzania

**Keywords:** Hypertensive crisis, Hypertensive emergency, Hypertensive urgency, Emergency Departments, Tanzania, Sub-Saharan Africa

## Abstract

**Background:**

Hypertensive crisis is among the causes of morbidity and mortality in adult patients with hypertension in Sub-Saharan Africa. We aimed to determine the burden, risk factors and describe the management strategies of hypertensive crisis among adult patients seen at emergency departments of district and regional hospitals in Tanzania.

**Methods:**

This was a prospective multicenter longitudinal study which included all 162 district and regional hospitals in Tanzania. It was part of the Tanzania Emergency Care Capacity Survey (TECCS), a large assessment of burden of acute illness and emergency care capacity in Tanzania. Adult patients who presented to emergency departments with blood pressure ≥ 180/110mmHg were enrolled. Demographics, clinical presentation, management, and 24-hours outcomes were recorded using a structured case report form. Descriptive statistics were summarized in frequency and median, while logistic regression was used to evaluate the association between risk factors and presence of hypertensive crisis.

**Results:**

We screened 2700 patients and enrolled 169 adults, henceforth proportion of adult patients with hypertensive crisis was 63 per 1000. Median age was 62 years (IQR 50–70 years) and predominantly females, 112 (66.3%). Majority 151(89.3%) were self-referred with two-wheel motorcycle being the commonest 46 (27.2%) mode of arrival to the hospital. Hypertensive emergency was found in over half 96 (56.8%) of the patients with hypertensive crisis, with oral medications administered in more than half of them, 71 (74%) as means to control the high blood pressure, and one-third 33 (34.4%) were discharged home. On multivariate analysis increasing age (AOR 4.53, p < 0.001), use of illicit drug (AOR 4.14, p-0.04) and pre-existing hypertension (AOR 8.1, p < 0.001) were independent risk factors for hypertensive crisis occurrence.

**Conclusion:**

Hypertensive crisis among adult patients attending district and regional hospitals is common (63 patients per every 1000 patients). Increasing age, use of illicit drug and pre-existing hypertension are independent associated factors for developing hypertensive crisis.

**Supplementary Information:**

The online version contains supplementary material available at 10.1186/s40885-023-00251-8.

## Background

Cardiovascular diseases are the leading cause of global morbidity and mortality [1], Africa has the highest prevalence of hypertension whereby 46% of the entire population over 25 years of age is estimated to be hypertensive [2]. As the incidence of hypertension increases globally, it is expected that more than 125 million people in sub-Saharan Africa (SSA) will be hypertensive by 2025 [3]. Untreated or poorly controlled high blood pressure (BP) also known as hypertension can lead to hypertensive crisis [4]. A hypertensive crisis can present without any associated symptoms in an individual, classified as hypertensive urgency [5, 6]. However, when it is associated with signs and symptoms of end-organ damage or failure it is termed hypertensive emergency [6].

It is well known that early detection and appropriate management of hypertension minimize complications [6], however, a significant number of patients in sub-Saharan Africa (SSA) present to hospitals when they already have developed signs and symptoms of end organ damage such as stroke, heart attacks, or kidney failure; which are the commonest cause of deaths among adults. Along with the high disease burden in SSA, optimal care models for diagnosing and treating hypertension in this region have not been well established.

In Tanzania, the existing regional and district health hospitals (equivalent to level II healthcare facilities) appear to be overwhelmed by the ever-increasing number of adult hypertensive patients. Hospitals at these levels of care are faced with the challenge of limited resources for the management of patient with hypertensive crisis [7, 8]. As a result most patients with complications of hypertension are referred to tertiary health facilities [9–11]. Therefore, we conducted a study to determine the burden of hypertensive crisis, its associated risk factors, and describe its management strategies among patients who present to emergency medicine departments of district and regional hospitals in Tanzania.

## Methods

### Study Design

This was a prospective multicenter longitudinal study of hypertensive adult patients conducted within 162 district and regional hospitals across Tanzania from 22 to 2020 to 03 February 2021. It was part of the Tanzania Emergency Care Capacity Survey (TECCS) which was a multicenter assessment of the burden of acute illness and emergency care capacity in country.

### Study setting

The healthcare system in the Tanzania consists of 162 regional and district hospitals which is equivalent to level II healthcare facilities. This study was conducted in emergency departments (ED) of **all 162 district and regional hospitals** in country. Patients are initially seen at the primary health facilities (dispensary and health center) before they are referred to district or regional hospitals. The EDs of these hospitals have treatment rooms designated for acutely ill patients but mostly they are not appropriately equipped for the purpose. They have some basic equipment to serve as outpatient clinics for stable patients. The medical staff consists of mainly general practitioner (medical officers, Assistant Medical Officers or clinical officers), Assistant Nurse Officers, and in a few hospitals’ emergency medicine specialists.

### Study participants

All adult patients aged 18 years and above presenting at ED of district and regional hospitals in Tanzania who had BP measurements taken on arrival. We excluded pregnant women, unconscious patients, and those who denied consent to participate in the study.

### Study protocol

Based on geographical proximity and easy accessibility of the districts, the visits were scheduled and executed within predetermined dates and time. This was a one-day survey therefore data were collected for 24 h per each study site. We screened all patients who had their BP measurements taken by healthcare providers at the hospital ED. Those who had BP ≥ 180/110 and had consented were enrolled into the study. Participants with one or more of the following: headache, acute visual loss, difficulty in breathing, chest pain, unilateral body weakness, altered mental status and reduced urine output were considered as hypertensive emergency and those without the mentioned symptoms were considered as hypertensive urgency. Socio-demographic, previous diagnosis of hypertension, compliance with hypertension medications, ED investigations, treatment, and disposition (admissions, discharge, death) were documented.

### Measurements

Blood pressure measurements were taken at the ED using a digital BP monitor (Medtech Novacheck BP – 09 N) and patients who were found to be hypertensive underwent a second blood pressure measurement using a manual sphygmomanometer (Spengler Lian Nano - sphygmomanometer Model 513,210) to confirm their readings. Digital BP machines and sphygmomanometers were provided by research assistants and BP measurements were preferentially completed in the sitting position, and supine position only for those patients who were unable to sit.

### Outcomes

**The primary outcome was 24 h mortality and secondary outcome** were factors associated with hypertensive crisis among patients who presented in hypertensive crisis at district and regional hospitals in Tanzania.

### Data analysis

From the structured case report form, data was transferred to Research Electronic Data Capture- REDCap (Version 9.1.21, Vanderbilt University, Nashville, TN, USA). It was then exported to Microsoft Excel where cleaning was done. A comprehensive data set was then exported to International Business Machines Statistical Package for Social Science (IBM SPSS) software for analysis. Percentages were calculated for categorical variables, and median with interquartile range (IQR) was calculated to summarize continuous variables. Logistic regression analysis was used to evaluate the association between risk factors and hypertensive crisis. Statistical significance was set at a p value of < 0.05.

## Results

**Fig. 1:**
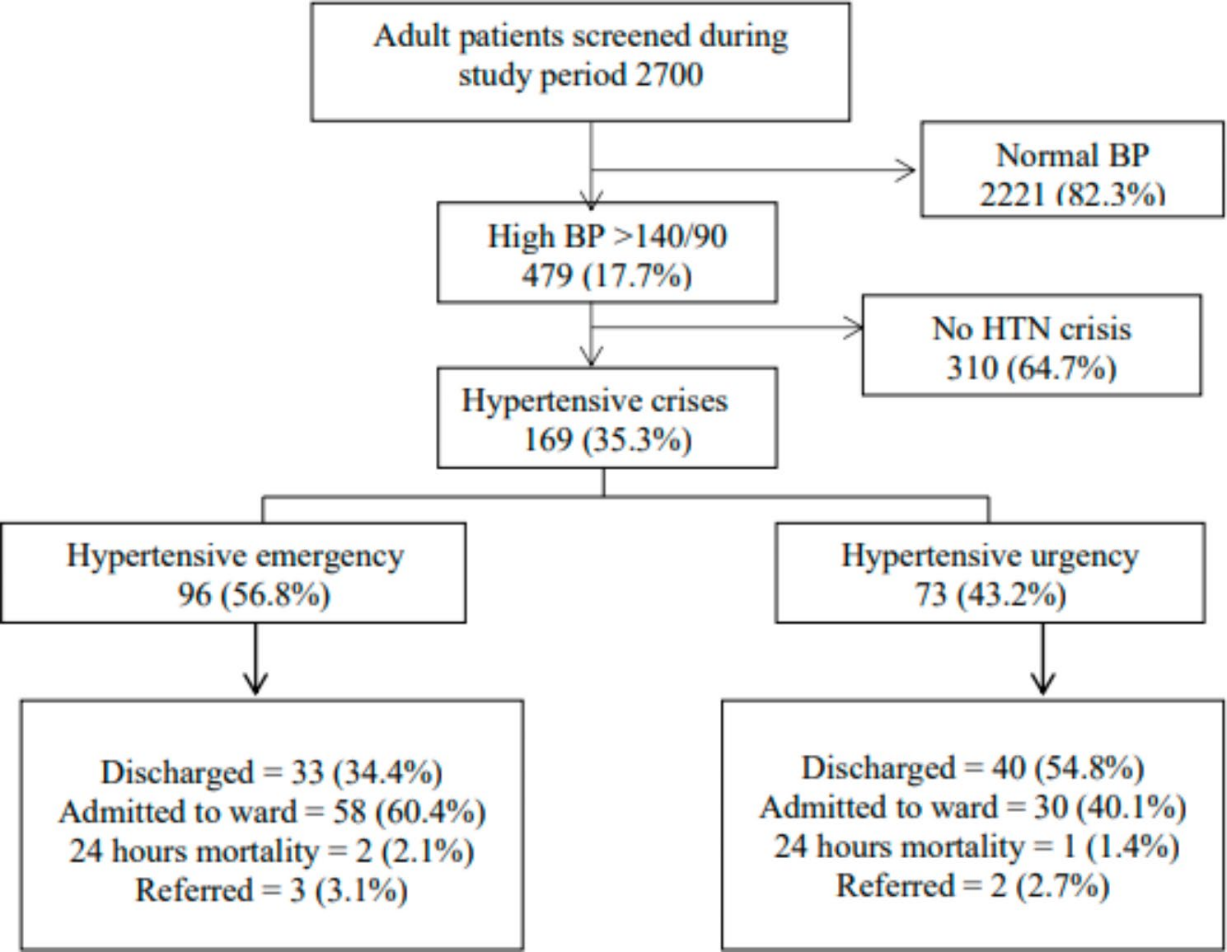
Patient distribution (disposition) during the study

Among 2700 patients who presented to the emergency departments during the study period, 479 (17.7%) had elevated blood pressure. Among those who had elevated BP (BP > 140/90 mmHg), 169 (35.3%) had hypertensive crises; in which 96 (56.8%) of them were hypertensive emergency and 73 (43.2%) were hypertensive urgency (Fig. 1). Therefore, the proportion of patients with hypertensive crisis among adult patients seen at district and regional hospitals was 6.3%.

### Socio-demographic characteristics

Among the 169 patients presenting with hypertensive crises, majority 78 (46.1%) were aged between 56 and 75 years, the median age was 62 years (IQR 50–70 years) and two-thirds, 112 (66.3%) were female. Most the patient were self-referred 151(89.3%) and the commonest modes of arrival to hospital were by two-wheeled motorcycles 46 (27.2%) followed by public transport 39(23%). Ambulances were used by a minority of them 7 (4.1%) (Table 1).

**Table 1:** Clinical characteristics of non-traumatic patients with hypertensive crisis

Variable		Hypertensive crisis (N = 169) n (%)	
**Sex**			
	Male	57 (33.7)	
	Female	112 (66.3)	
**Age in years**			
	18–35	15 (8.9)	
	36–55	46 (27.2)	
	56–75	78 (46.1)	
	Above 75	30 (17.8)	
Median age in years (IQR)		62(50–70)	
**Level of education**			
	University	10 (5.9)	
	Completed secondary school	13 (7.7)	
	Completed primary school	85 (50.2)	
	No formal education	54 (32.0)	
	Unknown	7 (4.2)	
**Referral status**			
	Referred	18 (10.7)	
	Self-referral	151 (89.3)	
**Mode of arrival**			
	Ambulance	7 (4,1)	
	Public transport	39 (23.1)	
	Private cars	25 (14.8)	
	Tricycle	31 (18.3)	
	Motorcycle	46 (27.2)	
	Walk in	15 (8.9)	
	Others	6 (3.6)	
**Presenting complains**			
	Headache	109 (64.4)	
	Short of breath	39 (22.9)	
	Chest pain	19 (11.5)	
	Altered mentation	14 (8.3)	
	Acute visual loss	12 (7.3)	
	Unilateral weakness	14 (8.3)	
**Exposure status**			
	History of cigarette smoking	4 (2.4)	
	History of alcohol consumption	24 (16)	
	Previous diagnosed with HTN	106 (62.7)	
	Substance (Drug) use	4 (2.4)	
	Not doing physical exercises	154 (91.1)	

### Clinical presentation and management

Study participants with hypertensive emergency presented with various clinical features; headache 62 (64.6%), difficulty in breathing 22 (22.9%), chest pain 11 (11.5%), altered mental status 8 (8.3%), and both unilateral body weakness and acute loss of vision each were in 7 (7.3%) participants.

Among patients with hypertensive emergency only 4 (4.2%) had electrocardiography (ECG), 8 (8.3%) had serum creatinine and 10 (10.4%) had chest x-rays performed. To control their BP at emergency department before disposition, oral route was the commonest mode of administration of medication in hypertensive emergency patients 71 (74%) with Nifedipine sublingual being the commonest medication given to 40 (41.7%) patients followed by Captopril 27 (28.1%). Intravenous (IV) antihypertensive were administered in only 25 (26%) patients with hypertensive emergency whereas 2 (2.7%) patients with hypertensive urgency. (Table 2)

**Table 2:** Management and outcome of patients with hypertensive crisis at district and regional hospitals in Tanzania

Management	Categories	Hypertensive emergency n (%)	Hypertensive urgency n (%)
Laboratory Investigation	Blood urea	8 (8.3)	6 (8.2)
	Cardiac maker	2 (2.1)	1 (1.4)
	Electrolytes	6 (6.3)	4 (5.5)
	Serum creatinine	8 (8.3)	5 (6.8)
Imaging	Chest X-rays	10 (10.4)	1 (1.4)
	CT scan of the brain	2 (2.1)	1 (1.4)
	Electrogram	4 (4.2)	3 (4.1)
	Fundoscopy	0 (0.0)	0 (0.0)
	MRI of the brain	0 (0.0)	0 (0.0)
	Ultrasound	1 (1.0)	0 (0.0)
Prescribed antihypertensive	Atenolol	4 (4.2)	6 (8.2)
	Captopril	27 (28.1)	23 (31.5)
	Furosemide	16 (16.7)	1 (1.4)
	Hydralazine	7 (7.3)	1 (1.4)
	Labetalol	1 (1.0)	0 (0.0)
	Losartan	19 (19.8)	6 (8.2)
	Nifedipine (sublingual)	40 (41.7)	33 (45.2)
	Nitroglycerin	1 (1.0)	0 (0.0)
	Nitroprusside	1 (1.0)	0 (0.0)
Route of administration	**Oral** antihypertensive	71 (74)	38 (52.1)
	IV antihypertensive No medication given	25 (26) 00 (0)	2 (2.7) 33 (45.2)
24 h Outcome	Admitted	58 (60.4)	30 (40.1)
	Died	2 (1.2)	1 (1.4)
	Discharged	33 (34.4)	40 (54.8)
	Referred	3 (3.1)	2 (2.7)

### Factors associated with hypertensive crisis

Logistic regression was done to ascertain risk factors for hypertensive crises. On multivariate analysis, increasing age, use of illegal/illicit drugs and pre-existing hypertension were found to be independently associated with the occurrence of hypertensive crisis. Being previously diagnosed with hypertension had a higher odd, AOR 8.10 (CI 95% 5.56–11.8, p-value < 0.001 than any other factors (Table 3).

**Table 3:** Factors associated with hypertensive crisis among hypertensive patient seen at district and regional level hospitals in Tanzania

	Univariate analysis			Multivariate analysis		
Variable	COR	95% CI	P -value	AOR	95% CI	P – value
**Age group (years)**						
36–55	4.64	2.57–8.37	**< 0.001**	2.66	1.38–5.11	**0.003**
56–75	10.89	6.21–19.10	**< 0.001**	3.65	1.88–7.08	**< 0.001**
≥ 75	15.05	7.91–28.64	**< 0.001**	4.53	2.10–9.84	**< 0.001**
18–35	Ref					
**Sex**						
Female	1.31	0.94–1.82	0.107	1.36	0.92–1.99	0.121
Male	Ref					
**Education level**						
A level secondary	0.26	0.03–2.09	0.206	0.70	0.08–6.00	0.743
O level secondary	0.36	0.15–0.86	**0.021**	0.45	0.18–1.15	0.095
Primary	0.94	0.48–1.85	0.854	0.72	0.34–1.56	0.408
None	2.03	1.01–4.09	**0.047**	1.11	0.49–2.53	0.799
Unknown	1.04	0.37–2.96	0.942	0.47	0.14–1.52	0.207
University	Ref					
**Alcohol consumption**						
Yes	1.23	0.79–1.92	0.366	1.17	0.68–2.00	0.569
No	Ref					
**Cigarette smoking**						
Yes	1.67	0.24–1.86	0.447	1.41	0.13–1.25	0.119
No	Ref					
**Substance abuse**						
Yes	1.84	0.64–5.25	0.255	4.14	1.06–16.13	0.041
No	Ref					
**Doing physical exercise**						
Yes	2.15	1.22–3.79	**0.008**	1.31	0.68–2.53	0.411
No	Ref					
**Previous diagnosed HTN**						
Yes	12.19	8.67–17.14	**< 0.001**	8.10	5.56–11.8	**< 0.001**
No	Ref					

Key: COR: crude odds ratio, AOR: adjusted odds ratio: Ref: Reference group, HTN: Hypertension

### Disposition of patients with hypertensive crisis

Among patients with hypertensive emergency, almost a third 33 (34.4%) of them were discharged home and 58 (60.4%) admitted for inpatient management. On 24 h follow up, mortality was 2 (2.1%) and 1 (1.4%) among hypertensive emergency and hypertensive urgency patients respectively. (Fig. 1)

## Discussion

At the emergency departments of district and regional hospitals in Tanzania we observed a high burden of hypertensive crisis among adult patients (63 per 1000 adult patients). Comparatively, it was evident that the proportion of patients with hypertensive emergency was slightly higher than those with hypertensive urgency. This is inconsistent with other scholars who found that the proportion of hypertensive urgency was higher than hypertensive emergency in patients with hypertensive crisis [12, 13]. Presence of a large proportion of patients with hypertensive emergency in this study can be explained by not only poor adherence to treatment and follow up plans but also inability to go for regular medical checkup among adult patients with hypertension, and the adult population in general. It is common practice among individuals in resource-limited settings to seek health care and evaluation only after realizing symptoms which suggest the development of illnesses. Yet even those few who attend regular follow up clinics have limited to no access to specialist physicians at these lower levels of care which eventually contributes to poor management of their hypertension.

In our study we found that patients in hypertensive crisis were not equally distributed with regards to gender, females constituted about two-third of the study population. Generally, in rural African communities, females tend to have better health-seeking behavior than males. Their male counterparts are usually overly occupied with bread-winning activities to provide for their families. Our study being hospital based it could easily include those who went to hospital to sick healthcare services, and majority were female participants.

Factors associated with occurrence of hypertensive crisis found in our study population do not differ from those reported in other studies [2, 9, 14]. Our study findings showed that previously diagnosed hypertension, increasing age, cigarette smoking and alcohol consumption are independently associated with hypertensive crisis. Although this might have something to do with one’s individual behavior which exposes them to the risk of hypertensive crisis, poor compliance can be due to problems with affordability and availability of appropriate antihypertensive medications in these lower-levels health facilities.

Like many other studies, increasing age was also found to be associated with being hypertensive hence predisposes an adult to the development of hypertensive crisis [3, 13, 15, 16]. Physiological changes such as loss of elasticity of blood vessels which accompany the normal aging process have been linked to the increased risk of developing hypertension in adults. The older population experiences reduced vitality, decreased level of physical activity, and financial constraints after retirement which to a large extent contribute to the limited access of care.

The management of hypertensive urgency and emergency varies in terms of choice of medication and its route of administration (Intravenous vs. oral by mouth). Hypertensive emergency requires rapid reduction of high blood pressure within one hour using intravenous antihypertensive medications, and not oral drugs. Our study found that very few patients with hypertensive emergency were treated by using intravenous antihypertensive medications which is against the existing guidelines and recommendations [7, 10, 17, 18]. The suboptimal management given could be attributed to limited availability of intravenous medications in the facilities, problems of affordability by clients and limited knowledge among healthcare providers on the current guidelines for management of hypertensive crisis [4].

Investigating the causes and complications of high blood pressure is among the core components of the management of hypertensive crisis especially hypertensive emergency. Our study observed that very few investigations were done to the patients with hypertensive crisis, which is not consistent with studies done in tertiary level Tanzania [19]. The differences can be attributed to; lack or limited availability of tests such as ECG, Echocardiogram, and other laboratory investigations. Moreover, even when the tests are available, skilled personnel to either order or correctly interpret the ordered test may not be there, and this poses a real obstacle to care.

The health care system in Tanzania is organized in such a way that district and regional hospitals are designated referral receiving facilities whose potential catchment areas are dispensaries and health centers. Geographically there is one hospital in each district and regional level of care. Patients may be required to travel long distances from remote areas, and some spend long periods of time on the way to these facilities. Many patients in our study reached the hospitals by public transport, and motorcycles were the commonest mode of transport. Such transport is unsafe to the patients who are already experiencing a true health emergency, hypertensive crisis. For patients who are already in hypertensive emergencies such as strokes and myocardial infarction whose managements are time sensitive and need continuation of care even during transportation appropriate choice and availability of transport is crucial. Certainly, the use of public transport and motorcycles to shuttle patients is risky and exposes them to other unforeseen incidents from road traffic accidents.

The local standard treatment guidelines instruct clinicians to formally refer patients who cannot be managed at the district hospitals to regional hospitals. Our study found that only few patients with hypertensive emergency were referred to regional hospitals despite the limited investigation and treatment options available at the district hospitals. In contrast, other studies have reported that facilities without medicines (antihypertensives), inadequate clinical investigations, and skilled staffs were seen to have more referrals to higher facilities [20]. The inappropriate decision not to refer these patients insinuates not only scarcity of resources but also poor knowledge of the management of hypertensive emergency at lower-level health facilities.

### Study strength

This study enrolled consecutive patients in all (162) district and regional hospitals in Tanzania. By its wide coverage of the facilities gives true representation of the situation.

### Study limitations

Collecting data for one day (24 h) at each hospital might not include daily or seasonal variation of flow of patients attending ED hence may have under or over-estimated some parameters. Also, we didn’t follow up to see the blood pressure changes at 24 h after admission, this could give details impact of the ED management strategies.

## Conclusion

The prevalence of hypertensive crisis among adult patients attending district and regional hospitals is high (63 patients per every 1000 patients) with increasing age, smoking, drug abuse and poor compliance to medication being independent associated factors for developing hypertensive crisis. We recommend regular clinical audits at the district and regional hospitals to ascertain resource capacity and delivery of evidence-based management of hypertensive crises and referral pathways.

### Electronic supplementary material

Below is the link to the electronic supplementary material.

Supplementary Material 1

## Data Availability

The dataset supporting the conclusion of this article is available from the authors on request.
