# Supplementary material for: The burden and management strategies of hypertensive crisis in adult patients presenting to emergency departments of district and regional hospitals in Sub-Saharan Africa
Source: Clin Hypertens. 2023 Oct 1;29:27. doi: 10.1186/s40885-023-00251-8 (PMC10544116; doi:10.1186/s40885-023-00251-8)
Supplement: Supplementary file 1 — Supplementary Material 1 [file 40885_2023_251_MOESM1_ESM.pdf]

RESPONSE TO REVIEWERS

SN	REVIEWER'S COMMENTS	REPOSNSE	PAGE NUMBER
	Reviewer 1 comments		
1	What is the term '100% capture' in the study setting? Does it mean that the authors studied all consecutive patients who visited emergency department?<br>The authors seem to emphasize that all subjects or sites follow the pre-planned protocol. It could be better to be more descriptive for the readers rather than using "100% capture".	Thank you for the comments,<br>The term "100% capture" meant managed to visit all district and regional hospitals in the country.<br>Following reviewer's concern, we removed the word 100% capture" and used "visited all district and regional hospitals".	2 & 4
2	In table 2, the number of performed lab tests and imaging were described. As this is not the management nor the outcome, that information could be deleted in the table.<br>> As the authors aim to give a contextual picture, what was the indication of running lab or imaging tests? Only a small portion of patients underwent those tests.	Thank you for the comment, The lab test was done at discretion of the attending doctor, we just reported our observations which indicated the practice in resource limited setting. Detailed description made in the discussion section that it can be due to lack or limited availability of tests such as ECG, Echocardiogram or skilled personnel to either order or correctly interpret the ordered test which pause obstacles to care.<br>We thought it is important to inform this to stimulate improvement in the care of these patients.	3
	Reviewer 2 comments		
1	Proportion of patients with hypertensive crisis" is an objective of this study, not an outcome	Thank you, we have corrected this	5
2	The sub-topics have to be shortened. (i.e., Socio-demographic characteristics of adult patients with hypertensive crisis at emergency departments of district and regional hospitals in Tanzania ----> Socio-demographic characteristics)	Thank you, this has been shortened	6 & 7
3	ECG---->electrocardiogram, Echocardiogram--->echocardiogram	Thank you, we corrected	6
4	In what order are the antihypertensive medications listed in Table 2? It doesn't appear to be in alphabetical order or organized by class.	Thank you, it is corrected in alphabetical order	16
